# Supplementary material for: Population Pharmacokinetic Analysis and Dosing Optimization of Prophylactic Fluconazole in Japanese Patients with Hematological Malignancy
Source: J Fungi (Basel). 2021 Nov 16;7(11):975. doi: 10.3390/jof7110975 (PMC8618010; doi:10.3390/jof7110975)
Supplement: Supplementary file 1 [file jof-07-00975-s001.zip › jof-1423684-supplementary.pdf]

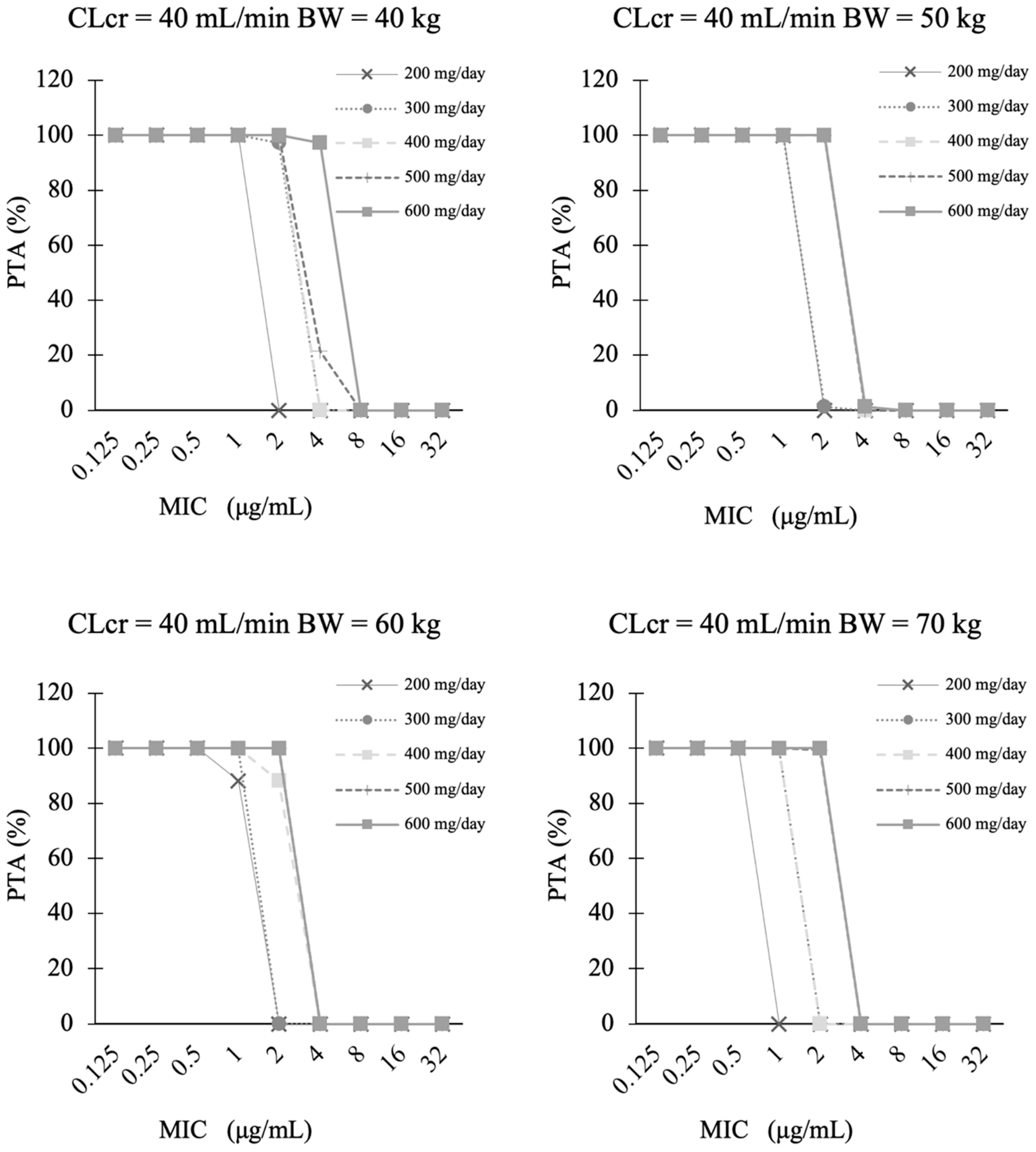

CLcr = 40 mL/min BW = 40 kg
CLcr = 40 mL/min BW = 50 kg
CLcr = 40 mL/min BW = 60 kg
CLcr = 40 mL/min BW = 70 kg
PTA (%)
MIC (μg/mL)
0.125
0.25
0.5
1
2
4
8
16
32
0
20
40
60
80
100
120
200 mg/day
300 mg/day
400 mg/day
500 mg/day
600 mg/day

Continued.

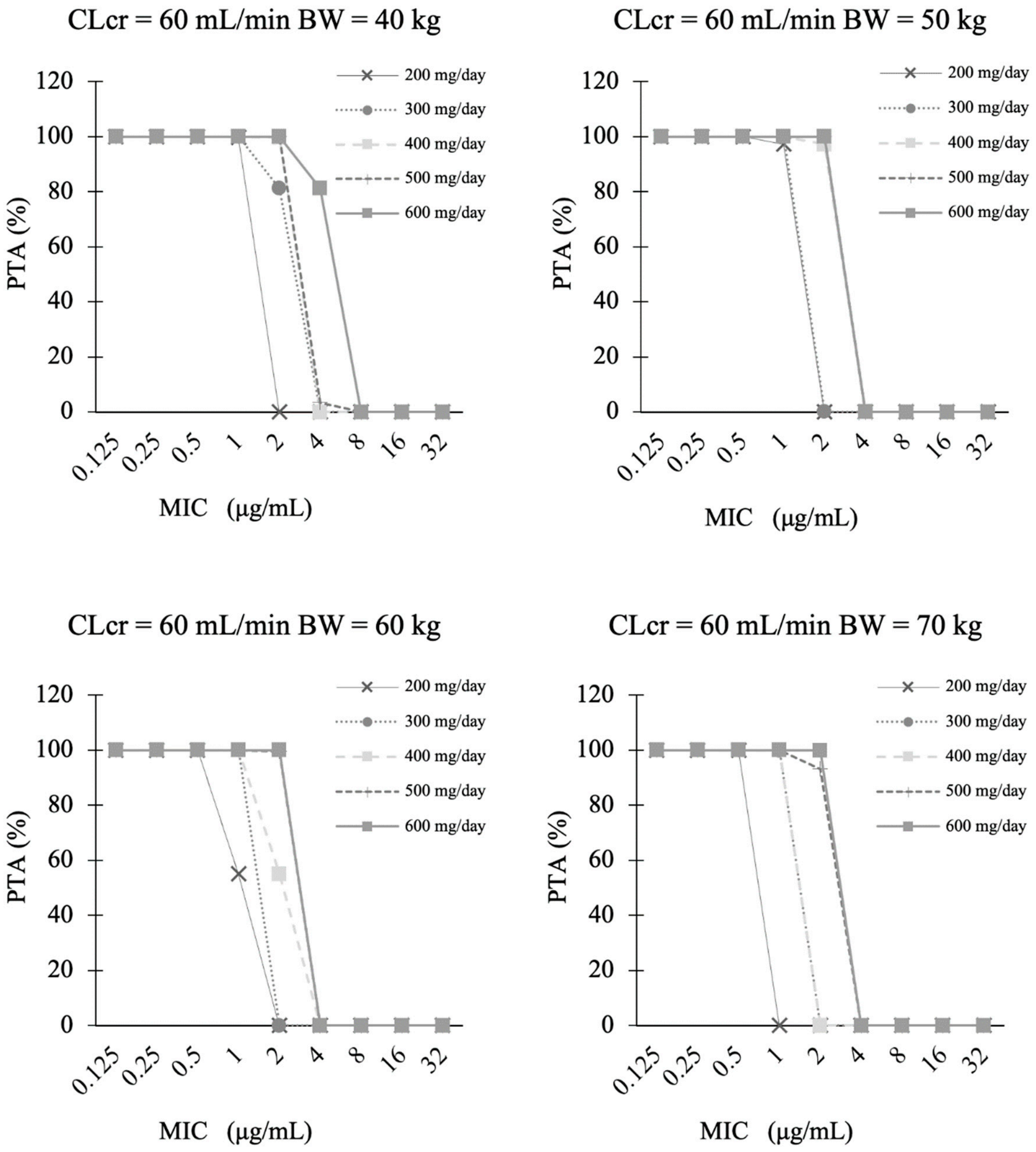
CLcr = 60 mL/min BW = 40 kg
CLcr = 60 mL/min BW = 50 kg
CLcr = 60 mL/min BW = 60 kg
CLcr = 60 mL/min BW = 70 kg
200 mg/day
300 mg/day
400 mg/day
500 mg/day
600 mg/day
PTA (%)
120
100
80
60
40
20
0
0.125
0.25
0.5
1
2
4
8
16
32
MIC (μg/mL)

Continued.

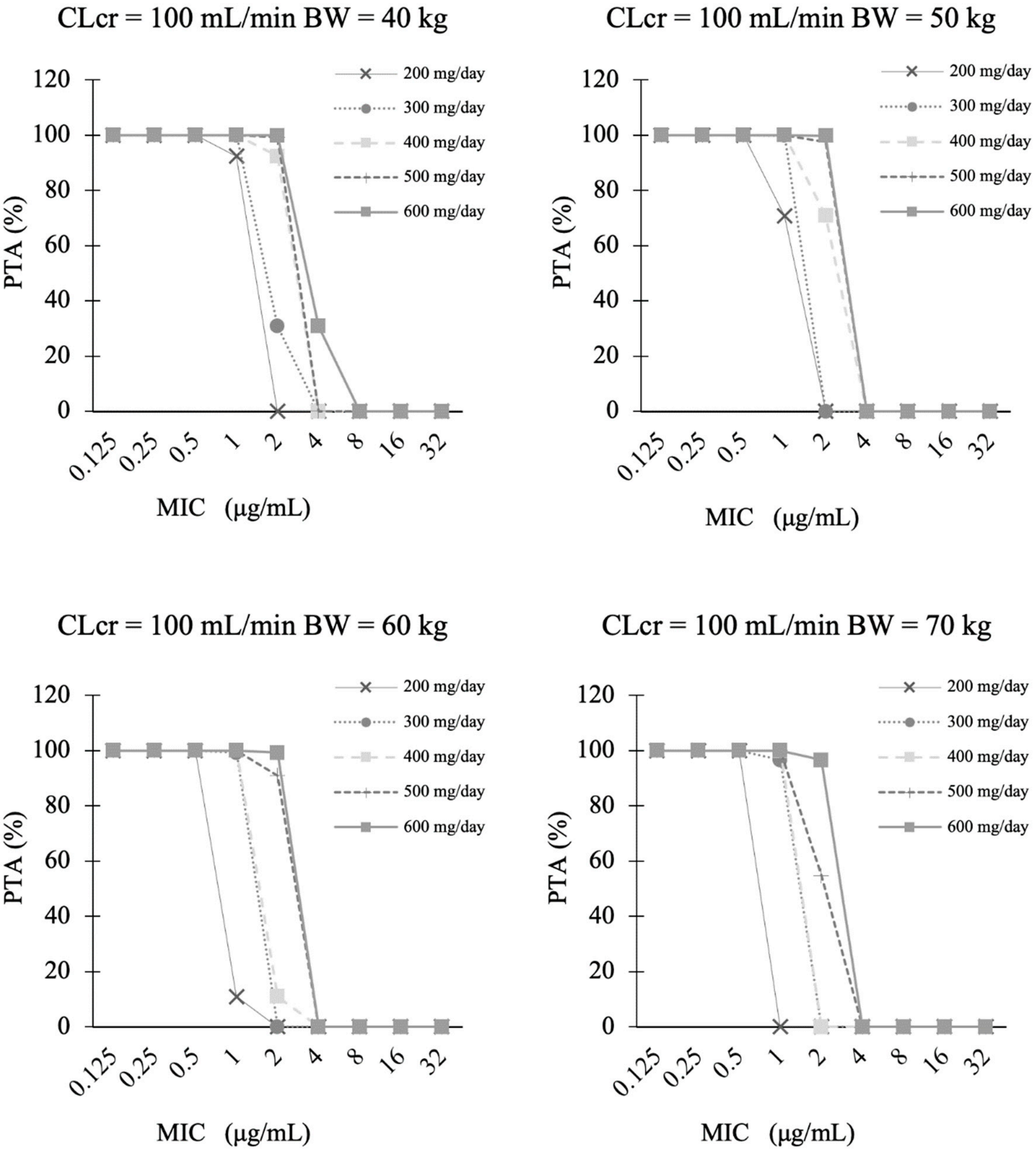
CLcr = 100 mL/min BW = 40 kg
CLcr = 100 mL/min BW = 50 kg
CLcr = 100 mL/min BW = 60 kg
CLcr = 100 mL/min BW = 70 kg
200 mg/day
300 mg/day
400 mg/day
500 mg/day
600 mg/day
PTA (%)
MIC (μg/mL)
0
20
40
60
80
100
120
0.125
0.25
0.5
1
2
4
8
16
32

Continued.

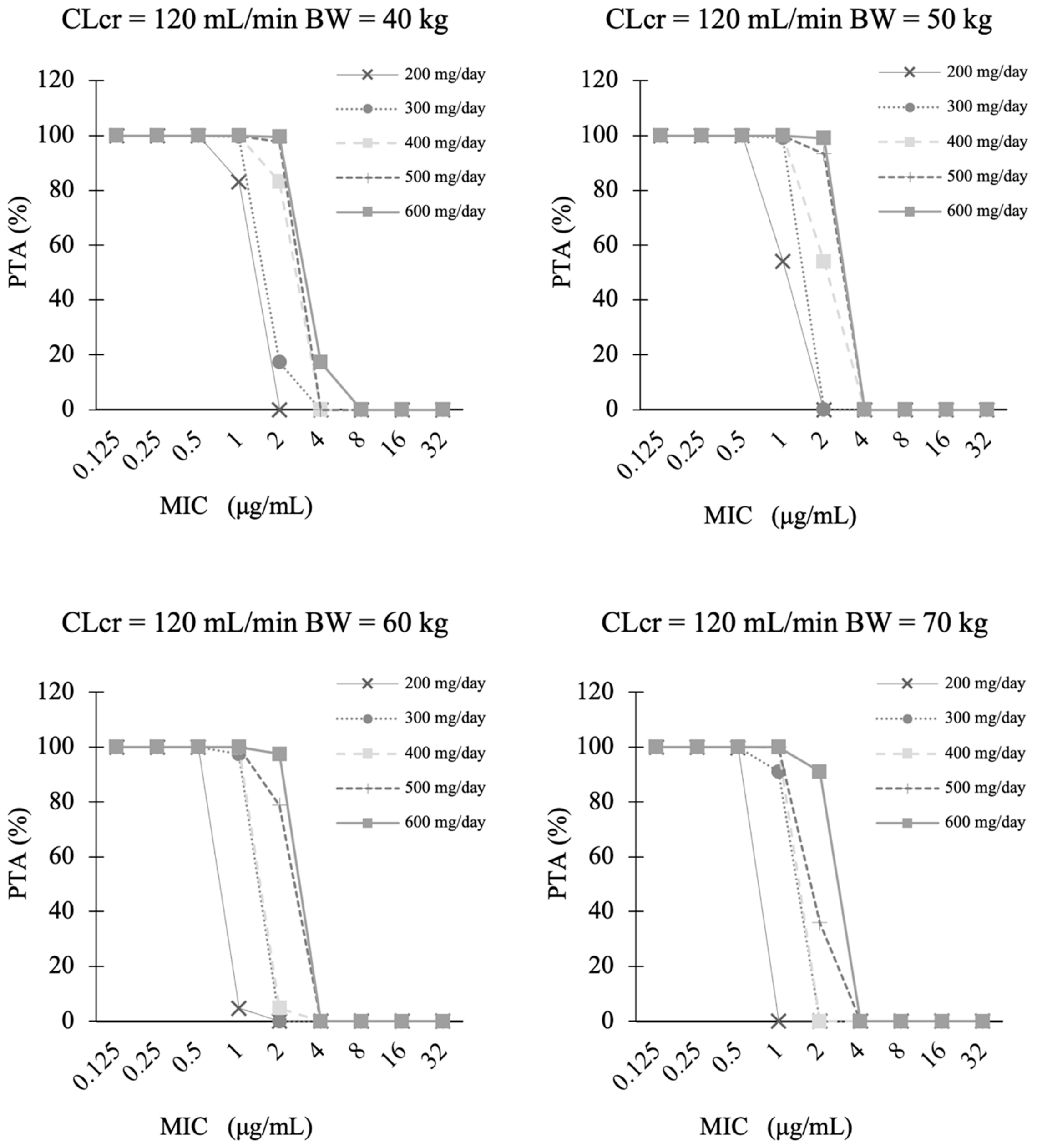
CLcr = 120 mL/min BW = 40 kg
CLcr = 120 mL/min BW = 50 kg
CLcr = 120 mL/min BW = 60 kg
CLcr = 120 mL/min BW = 70 kg
200 mg/day
300 mg/day
400 mg/day
500 mg/day
600 mg/day
PTA (%)
MIC (μg/mL)
0.125
0.25
0.5
1
2
4
8
16
32
0
20
40
60
80
100
120

Continued.

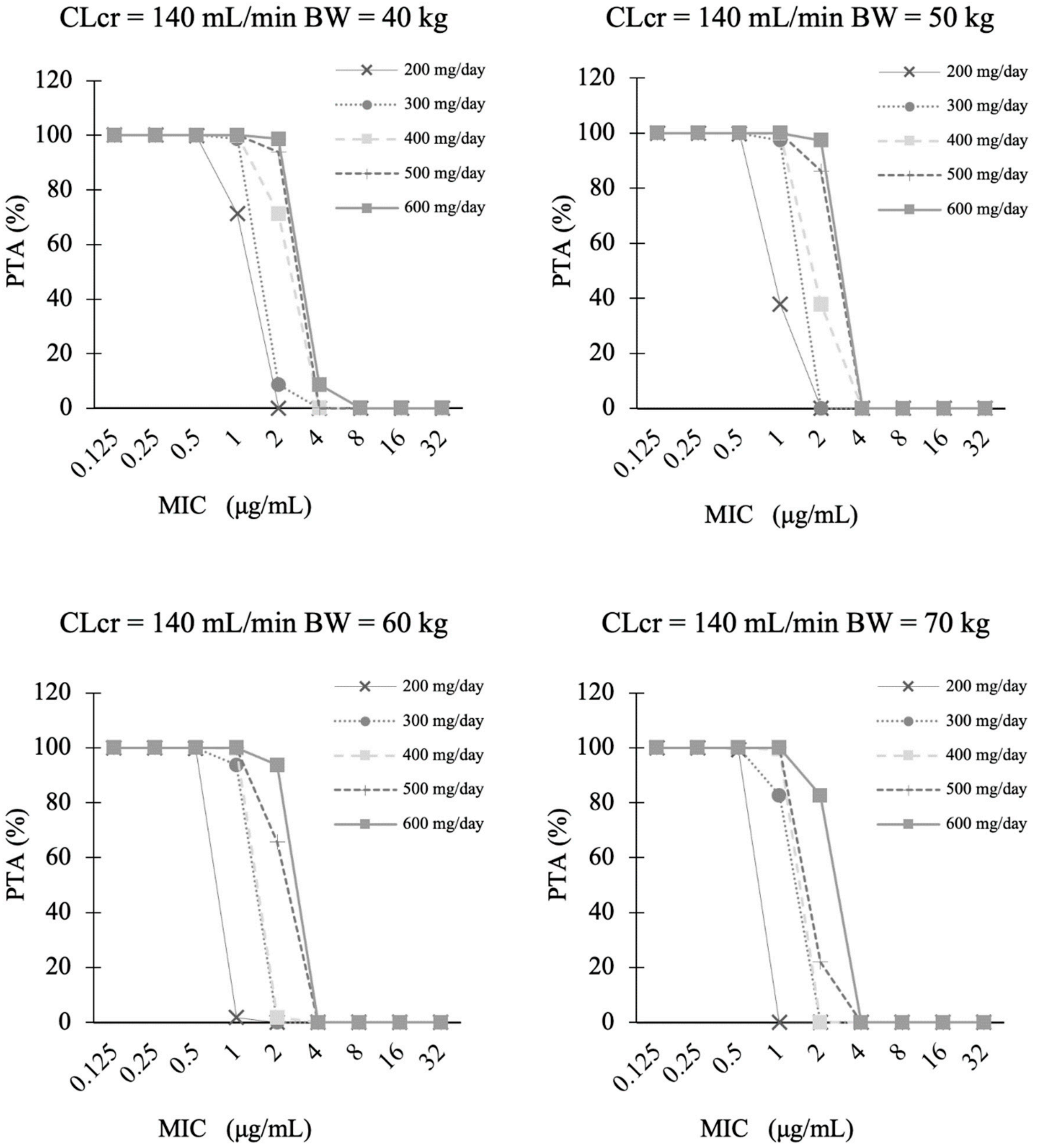

Figure S1. Probability of target attainment (PTA) of *f* AUC/ MIC ratio ≥ 50 after fluconazole loading doses of 200mg, 300mg, 400mg, 500mg, and 600 mg administered on Day1 (0–24hr). Target MIC is 2 μg/mL, and CLcr (Cockcroft-Gault equation) is 40, 60, 100, 120, and 140 mL/min. CLcr 80mL/min is shown in Fig 3.

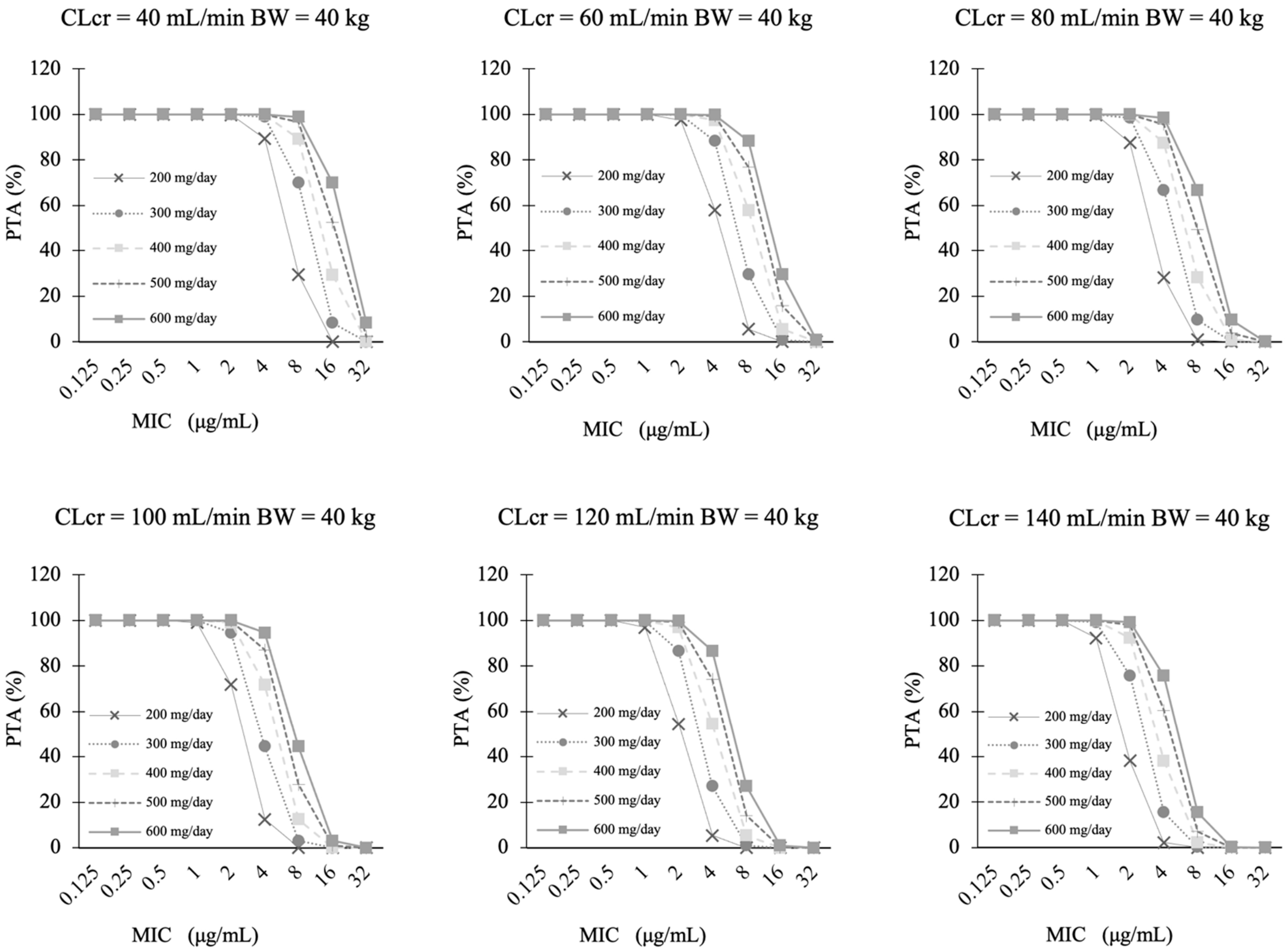
CLcr = 40 mL/min BW = 40 kg
CLcr = 60 mL/min BW = 40 kg
CLcr = 80 mL/min BW = 40 kg
CLcr = 100 mL/min BW = 40 kg
CLcr = 120 mL/min BW = 40 kg
CLcr = 140 mL/min BW = 40 kg
PTA (%)
MIC (μg/mL)
200 mg/day
300 mg/day
400 mg/day
500 mg/day
600 mg/day
0.125
0.25
0.5
1
2
4
8
16
32

Continued.

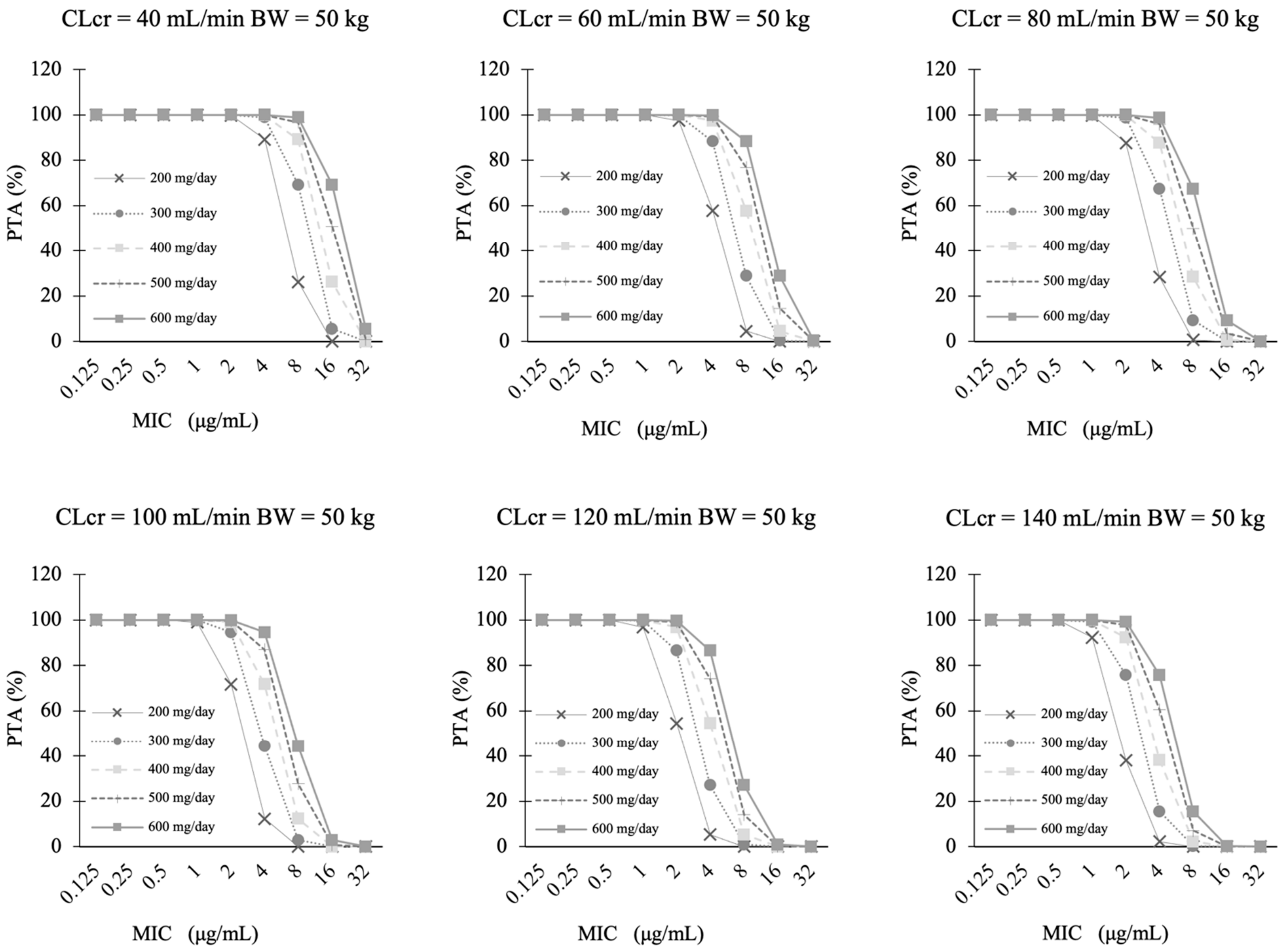

CLcr = 40 mL/min BW = 50 kg
CLcr = 60 mL/min BW = 50 kg
CLcr = 80 mL/min BW = 50 kg
CLcr = 100 mL/min BW = 50 kg
CLcr = 120 mL/min BW = 50 kg
CLcr = 140 mL/min BW = 50 kg
PTA (%)
MIC (μg/mL)
0.125
0.25
0.5
1
2
4
8
16
32
0
20
40
60
80
100
120
200 mg/day
300 mg/day
400 mg/day
500 mg/day
600 mg/day

Continued.

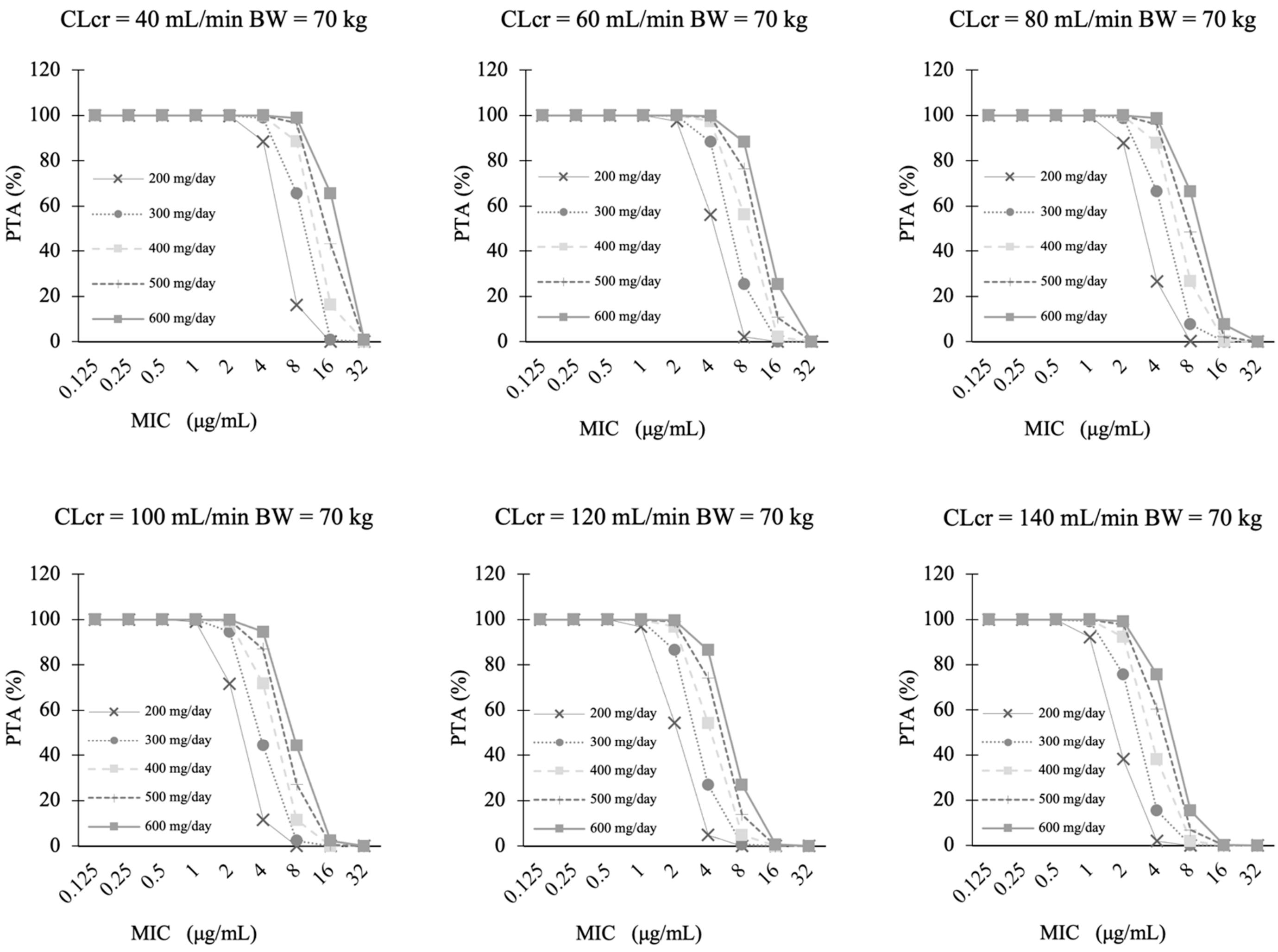

Figure S2. Probability of target attainment (PTA) of *f* AUC/ MIC ratio ≥ 50 after fluconazole maintenance doses of 200mg, 300mg, 400mg, 500mg, and 600 mg administered on Day15 (336–360hr). Target MIC is 2 μg/mL, and Body Weight is 40, 50, and 70 kg. Body Weight 60 kg is shown in Fig 4.
